# ALK阳性非小细胞肺癌靶向治疗研究进展

**DOI:** 10.3779/j.issn.1009-3419.2014.12.05

**Published:** 2014-12-20

**Authors:** 丽 马, 树才 张

**Affiliations:** 101149 北京，首都医科大学附属北京胸科医院，北京市结核病胸部肿瘤研究所肿瘤内科 Department of Medical Oncology, Beijing Chest Hospital, Capital Medical University, Beijing Tuberculosis and Thoracic Tumor Research Institute, Beijing 101149, China

**Keywords:** ALK, 肺肿瘤, 克唑替尼, ALK, Lung neoplasms, Crizotinib

## Abstract

*ALK*基因重排在非小细胞肺癌（non-small cell lung cancer, NSCLC）中发生率约3%-5%。第一代ALK酪氨酸激酶抑制剂Crizotinib可以有效地针对这些肿瘤在NSCLC个体化靶向治疗中发挥重要作用。随机Ⅲ期临床研究对比了Crizotinib和二线标准化疗方案治疗ALK阳性晚期NSCLC患者的疗效，提示Crizotinib治疗ALK阳性的晚期NSCLC患者疗效明显、耐受性好。然而，尽管治疗初期有较好的疗效，但大部分患者持续用药后会出现获得性耐药。因此，一些针对ALK阳性的NSCLC患者的新治疗策略在临床试验中逐步开展，包括第二代ALK抑制剂，比如LDK378、CH5424802（RO5424802）和AP26113等，以及其他靶点抑制剂如热休克蛋白90抑制剂等。本文就ALK阳性NSCLC患者的治疗进展以及最新药物临床研究做一综述。

## Abstract (en)

The rate of the anaplastic lymphoma kinase (*ALK*) gene rearrangements in non-small cell lung cancer (NSCLC) tissues is 3%-5%. The first-in-class ALK tyrosine kinase inhibitor, crizotinib, can effectively target these tumors represent a significant advance in the evolution of personalized medicine for NSCLC. A randomized phase Ⅲ clinical trial in which superiority of crizotinib over chemotherapy was seen in previously treated ALK-positive NSCLC patients demonstrated durable responses and well tolerance in the majority of ALK-positive NSCLC patients treated with crizotinib. However, despite the initial responses, most patients develop acquired resistance to crizotinib. Several novel therapeutic approaches targeting ALK-positive NSCLC are currently under evaluation in clinical trials, including second-generation ALK inhibitors, such as LDK378, CH5424802 (RO5424802), and AP26113, and new agents shock protein 90 inhibitors. This review aims to present the current knowledge on this fusion gene, the treatment advances, and novel drug clinical trials in ALK rearranged NSCLC.

随着分子医学研究进展及靶向药物的不断涌现，非小细胞肺癌（non-small cell lung cancer, NSCLC）的治疗已进入个体化诊疗时代。间变性淋巴瘤激酶（anaplastic lymphoma kinase, *ALK*）基因重排是NSCLC中新的肿瘤驱动基因^[1]^。针对*ALK*融合基因的小分子抑制剂克唑替尼（Crizotinib）是一种ATP竞争性酪氨酸酶抑制剂。2011年，美国国立综合癌症网络（National Comprehensive Cancer Network, NCCN）和欧洲肿瘤医学会（European Society for Medical Oncology, ESMO）同时推荐ALK阳性NSCLC一线治疗首选克唑替尼^[2]^。相继，在2013年初，国家食品药品监督管理总局（China Food and Drug Administration, CFDA）也已批准克唑替尼用于治疗局部晚期或转移性ALK阳性NSCLC。然而，尽管克唑替尼在ALK阳性患者获得良好中位无进展生存期（progression-free survival, PFS）和客观有效率（objective response rate, ORR），最终还是难逃耐药而治疗失败的命运^[3, 4]^。本文阐述了ALK阳性NSCLC的生物学临床特征，概述了靶向ALK的治疗疗效、耐药机制，并探讨了针对*ALK*融合基因的新的靶向治疗，为今后临床应用提供依据。

## 1 *ALK*和*ALK*融合基因的生物学特性

*ALK*基因定位于2号染色体的短臂，可编码胰岛素受体超家族中的一个1, 620个氨基酸的受体酪氨酸激酶。ALK蛋白由胞外联接区、跨膜区、胞内酪氨酸激酶区组成。目前，ALK的生理功能并不清楚^[5]^。NSCLC *ALK*基因的激活是由ALK染色体重排导致，即ALK酪氨酸激酶区与5'末端棘皮动物微管结合蛋白（echinoderm microtubule-associated protein-like 4, EML4）形成融合基因。5'末端的融合基因发生低聚反应，使ALK酪氨酸激酶不依赖配体而激活，从而导致下游通路激活，如Ras/MAPK、PI3K/AKT和JAK/STAT信号通路等^[6]^。

在细胞系和动物模型实验中，ALK驱动的肺癌对ALK抑制剂有良好的反应，在ALK抑制剂作用下，肿瘤也可逐渐缩小^[7]^。这一发现提示“癌基因依赖”（oncogene addiction）现象，肿瘤逐渐依赖这种肿瘤驱动基因而生长，因此对该驱动基因的抑制剂具有高敏感性。

迄今为止，NSCLC中已鉴定出约14种EML4-ALK变异体，而这些不同的变异体是否具有不同的生物学特性目前仍不清楚。体外研究提示三种EML4-ALK变异体对小分子ALK抑制剂的敏感性存在差异，可能与融合基因稳定性有关^[8]^。然而，在临床研究中，并未报道不同EML4-ALK变异体对crizotinib的ORR存在差异。

## 2 ALK在NSCLC中的特点

2007年两个独立的研究小组同时发现了NSCLC中存在*ALK*基因重排，发生率约3%-5%^[1, 9]^。ALK阳性的NSCLC多为年轻腺癌、不吸烟或少吸烟患者，与一些特殊的形态学特征密切相关，如印戒细胞癌或粘液筛状癌，多数表达甲状腺转录因子-1和p63^[9]^。*ALK*基因重排常常与*EGFR*或*KRAS*突变相斥^[10]^。由于ALK阳性NSCLC患者与存在其他分子改变的患者临床病理特征存在相似之处，如*EGFR*突变、*ROS1*和*RET*基因重排，因此，其分子特征需要进一步准确鉴定才能在临床实践中具有明确指导意义。

此外，ALK阳性患者对含铂双药化疗的疗效与其他患者并无明显差异^[11]^。然而，值得一提的是，Ⅲ期临床研究提示，*ALK*融合基因阳性患者对单药培美曲塞有较高的ORR（29%），而非选择的NSCLC患者ORR只有10%^[12]^。同时，EGFR酪氨酸激酶抑制剂对ALK阳性患者是无效的。

## 3 第一代ALK抑制剂Crizotinib及其耐药

Crizotinib（Xalkori, PF-02341066; Pfizer）是一个选择性ATP竞争性小分子口服ALK、c-MET/肝细胞生长因子受体和ROS1受体酪氨酸激酶抑制剂。Crizotinib在ALK阳性的晚期NSCLC患者的临床疗效研究已经相继开展。

首个研究（PROFILE 1001）Ⅰ期试验扩展组中将接受Crizotinib治疗的ALK阳性复发NSCLC患者82例和ALK阳性未使用Crizotinib治疗对照组23例进行比较，结果显示二、三线Crizotinib治疗组1年和2年生存率分别为70%、55%，高于ALK对照组的44%、12%（*P*=0.02）。表明ALK阳性的晚期NSCLC患者二、三线接受Crizotinib治疗的总生存时间明显长于未接受Crizotinib治疗组以及ALK阴性对照组^[13]^。单臂研究募集了119例接受Crizotinib治疗的ALK阳性患者，其总的中位治疗持续时间为32周^[13]^。其中，2例完全缓解，69例部分缓解（ORR达到61%），有效患者的中位治疗持续时间为48周。这些结果最近被更新为包括143例ALK阳性的NSCLC患者，ORR仍为61%，中位有效治疗持续时间为49周，中位PFS为9.7个月^[14]^。另一个单臂研究（PROFILE 1005）募集了136例患者接受Crizotinib治疗，总的中位治疗持续时间为22周，其中1例完全缓解，67例部分缓解（ORR达到50%），中位有效持续治疗时间为47周。最新结果更新为261例ALK阳性的NSCLC患者^[14]^。中位有效持续治疗时间为48周，ORR达到60%，中位PFS达到8.1个月。这两项研究成为FDA快速批准Crizotinib治疗晚期ALK阳性NSCLC的基础。随机Ⅲ期临床研究（PROFILE 1007）对比观察了Crizotinib和二线标准化疗方案治疗曾接受过一线含铂双药治疗后进展的*ALK*融合基因阳性NSCLC患者的疗效，Crizotinib组的PFS比化疗组延长两倍以上，173例随机接受Criozitnib治疗患者的中位PFS为7.7个月，而随机接受化疗的174位患者中位PFS为3个月^[11]^。与PFS的结果一致，Crizotinib组的有效率超过化疗组的三倍，Crizotinib组的ORR为65%，而化疗组为19%，培美曲塞和多西他赛组ORR分别为29%和7%。此外，今年的美国临床肿瘤学会（American Society of Clinical Oncology, ASCO)大会中报道了Crizotinib与培美曲塞联合顺铂或者卡铂一线治疗ALK阳性晚期非鳞NSCLC患者疗效对比的Ⅲ期临床研究结果（PROFILE 1014）。Crizotinib疗效显著优于化疗，ORR分别为74%和45%，PFS分别为10.9个月和7.0个月。另外，有68%的患者仍处于随访中，OS结果目前尚未显示出显著的改善（HR=0.821, *P*=0.180），为Crizotinib一线治疗ALK阳性NSCLC又添新证^[15]^。

Crizotinib的Ⅰ期、Ⅱ期和Ⅲ期研究^[11]^中，报道的副反应主要包括视力障碍、腹泻、恶心、呕吐、浮肿、便秘及转氨酶升高等。最常见的Crizotinib相关的3级-4级药物副反应为中性粒细胞减少和转氨酶水平的升高。

尽管Crizotinib初始反应良好，但大部分患者在治疗1年内耐药出现疾病复发，被称为获得性耐药^[3]^。Crizotinib的获得性耐药机制被分为两类，第一类包括遗传靶点基因变异，如*ALK*突变或基因扩增。首个Crizotinib获得性耐药的报道中，将一例接受Crizotinib治疗复发后的腹水标本深度测序，结果提示两个不重叠的ALK酪氨酸激酶区域的突变，L1196M和C1156Y。体外实验提示以上任何一种突变都可以导致Crizotinib的耐药。随后，其他ALK激酶区域的耐药突变在患者样本中陆续被发现，如G1269A、1151Tins、L1152R、1202R和1206Y。此外，*ALK*融合基因扩增在体内外已经被鉴定为耐药的原因。总之，大约三分之一的患者接受Crizotinib治疗过程中复发是由于ALK耐药突变或*ALK*融合基因的扩增^[16]^。

第二种耐药机制为其他ALK相关信号通路的激活^[4]^。体外研究提示，EGFR和其他HER家族受体酪氨酸激酶能够通过配体介导激活ALK受体而导致耐药。此外，*C-KIT*基因扩增也可导致Crizotinib耐药。其他潜在旁路机制包括*EGFR*和*KRAS*的激活突变等。

## 4 第二代ALK抑制剂

### 4.1 LDK378

LDK378（Ceritinib; Novartis pharmaceuticals）是一种新的强效选择性小分子酪氨酸激酶抑制剂，靶向ALK。LDK378是在TAE684基础上合成的，TAE684是一种早期合成的ALK抑制剂。生物化学分析中，LDK378对ALK选择性更高。EML4-ALK阳性的H2228细胞系裸鼠模型中，LDK378治疗导致剂量依赖性肿瘤抑制，不同剂量中耐受性良好。一项国际多中心Ⅰ期临床研究^[17]^评价了LDK378的临床疗效。LDK378治疗晚期ALK阳性NSCLC有效率高。最新研究^[17]^表明，LDK378对大多数Crizotinib抵抗的ALK阳性患者有效率较高，其中也包括从未用药治疗的患者，并且大多数不良反应较轻。在这项研究的扩展阶段试验，在130例肿瘤患者中评估了LDK378的抗肿瘤活性、安全性和药代动力学，这些患者中114例每天至少应用LDK378 400 mg，疾病缓解率为58%（95%CI: 48%-67%），中位PFS为7个月（95%CI: 5.6-9.5）。对于80例以前接受过Crizotinib治疗的患者中，疾病缓解率达56%（95%CI: 45%-67%）^[18]^。基于上述研究，Ceritinib（LDK378）于2014年4月30日获得美国食品药物管理局（Food and Drug Administration, FDA）批准用于*ALK*融合基因阳性晚期NSCLC的治疗。今年ASCO大会报道了在克唑替尼治疗ALK阳性的晚期NSCLC患者疾病进展后，Ceritinib治疗的有效率可达到55%-70%，PFS可达6.9个月-7个月，Ceritinib治疗也可显示出较强的脑部抗肿瘤活性^[19]^。此外，研究^[20]^还显示Ceritinib治疗对ALK+的亚裔和高加索NSCLC患者均有抗肿瘤活性和良好的耐受性，为*ALK*融合基因阳性晚期NSCLC患者克唑替尼治疗耐药后的治疗提供了一种新的选择。

### 4.2 CH5424802

CH5424802（RO 5424802/Alectinib; Chugai pharmaceuticals和Roche pharmaceuticals）是一种强效选择性苯并咔唑ALK抑制剂，对于ALK和ALK酪氨酸激酶区域突变（包括L1196M突变、F1174L和R1275Q突变等）均有效^[21]^。Ⅰ期临床试验（AF-002JG）中，24例患者接受了300 mg-900 mg每天两次的剂量爬坡试验，初步缓解率达到54.5%^[22]^。推荐最大耐受剂量为600 mg，每天两次，此剂量被推荐到Ⅱ期临床研究中。在Ⅱ期临床研究中，进行评价的46例患者有93.5%（43/46）的患者获得客观有效（2例完全缓解和41例部分缓解），患者中位持续治疗时间至少14个月，临床用药后患者起效时间较快，65%（30/46）的患者在3周内达到部分缓解，87%（40/46）的患者在6周内达到部分缓解^[21]^。3例脑转移的患者也可以观察到临床疗效。安全性评价具有良好耐受性，最常见副反应为1级味觉障碍（30%）、转氨酶短时升高（28%）、胆红素增加（30%）和皮疹（28%）。初步临床数据提示能显著抑制中枢神经系统转移，该药已经获得FDA突破性药物认证。Alectinib用于治疗克唑替尼耐药的ALK阳性NSCLC患者的全球单臂Ⅱ期研究已经启动（Accalia G2L, NP28673, NCT01801111）。

### 4.3 AP26113

AP26113是新一代的ALK抑制剂，其对ALK酪氨酸激酶继发性突变L1196M和其他*ALK*耐药突变均有效，对ROS1和EGFR T790M突变的患者也有效。今年ASCO更新了正在进行的Ⅰ期/Ⅱ期临床研究（NCT01449461）数据，提示其在ALK阳性NSCLC患者中抗肿瘤效果显著，ALK阳性患者中，63%（15/24）达到客观有效（1例完全缓解和14例部分缓解），此外，对75%（12/16）Crizotinib耐药的患者也有效，重要的是，5例脑转移的患者中4例有效^[23]^，常见的副反应为恶心（33%）、乏力（22%）和腹泻（20%）。一些其他新一代ALK抑制剂也在陆续进入临床研究，如X-396（Xcovery）（NCT01625234）、ASP 3026（Astellas Pharma）（NCT01284192）和TSR-011（Tesaro）等。

## 5 热休克蛋白90（heat shock protein 90, HSP90）抑制剂

*EML4-ALK*融合基因被发现是HSP90的客户蛋白。在*EML4-ALK*融合基因的细胞系研究中，HSP90抑制剂（如17-AAG、17-DMAG、AUY922以及Ganetespib）可降低ALK融合蛋白的表达水平，抑制肿瘤细胞生长。一项Ganetespib Ⅱ期临床研究^[24]^中，8例*ALK*基因重排患者中，有4例部分缓解，3例病变稳定。对Crizotinib耐药的ALK阳性NSCLC患者中，1例患者对Ganetespib治疗达到客观有效。此外，AUY922（Novartis）在单药治疗接受或未接受过Crizotinib治疗的ALK阳性NSCLC患者中均具有临床活性。最新美国一项非随机开放多中心Ⅱ期临床研究^[25]^提示，Ganetespib在经多程治疗且无*EGFR*和*KRAS*突变晚期NSCLC患者有明确疗效，尤其是在*ALK*基因重排患者中有效率达50%（NCT01031225）。此外，临床前研究提示ALK抑制剂和HSP90抑制剂具有一定的协同作用。一些联合治疗的研究正在进行中，如Crizotinib联合Ganetespib（NCT01579994）、Crizotinib联合AT13387（NCT01712217）和LDK378联合AUY922（NCT01772797）。

今年的ASCO大会上，除了上述药物研究更新数据外，新的ALK抑制剂也有报道。比如，作为ALK原肌球蛋白受体激酶抑制剂，Ⅰ期/Ⅱ期临床研究^[26]^显示，TSR-011可以有效抑制Crizotinib耐药的ALK阳性NSCLC（NCT02048488）。X-396作为第二代ALK抑制剂，在克服Crizotinib耐药方面也显示了活性^[27]^。此外，在*ALK*融合基因检测方法上也进行了探讨，包括胸腔积液、细针穿刺和粗针穿刺活检组织的检测意义，以及PCR、IHC和FISH检测的方法学和经济学评价，为今后临床应用奠定了基础^[28]^。

## 6 结语

NSCLC中，*ALK*基因重排的发现和针对其靶向治疗的研究在其个体化治疗过程中是一个重要的里程碑。值得一提的是从*ALK*基因重排在NSCLC中鉴定（2007年）到FDA批准其抑制剂Crizotinib用于临床实践（2011年）经历了仅仅4年时间。*ALK*基因重排的检测和针对ALK阳性患者的治疗目前已经成为标准化的治疗方案。尽管初始反应疗效显著，但大部分患者会发生获得性耐药。分析ALK耐药机制并进一步改进治疗策略可能克服耐药的发生。新的强效ALK抑制剂和HSP90抑制剂在这方面均具有一定活性。关键的问题包括如何选择这些药物、如何制定最优的治疗方案、怎样更好的联合用药并减少毒性等，还需要大量的临床研究进一步明确，从而在ALK阳性的NSCLC患者中达到长期的疾病控制。
